# Recent advances in bronchoscopy

**DOI:** 10.12688/f1000research.14596.1

**Published:** 2018-10-16

**Authors:** Elaine Dumoulin

**Affiliations:** 1 Department of Medicine, University of Calgary, Calgary, Canada

**Keywords:** Bronchoscopy, advancement, technology, interventional pulmonary medicine

## Abstract

Bronchoscopy is a very common tool for diagnosis and therapeutic purposes in dealing with diseases of the lungs and the airways. Thankfully, a multitude of new technologies have made it more accessible for the use of physicians. This article is a review of the indication of bronchoscopy as it is being used today for a variety of chest pathologies.

Bronchoscopy has come a long way. Designed at first as a rigid metal pipe to assist in foreign body removal, it has become a very common tool for diagnosis and therapeutic purposes when dealing with diseases of the lungs and the airways. With the option of a rigid (as pictured in Figure 1) or flexible bronchoscope and advancements in specific and precise tools, the science of bronchoscopy has evolved greatly. Tools include cold, heat, balloons, valves, and stents. Bronchoscopy is used in cancer, interstitial lung disease, vascular disease, foreign body removal, and chronic obstructive pulmonary disease management, to name just a few applications. This article is a review of the indication of bronchoscopy as it is being used today for a variety of chest pathologies.

**Figure 1.**
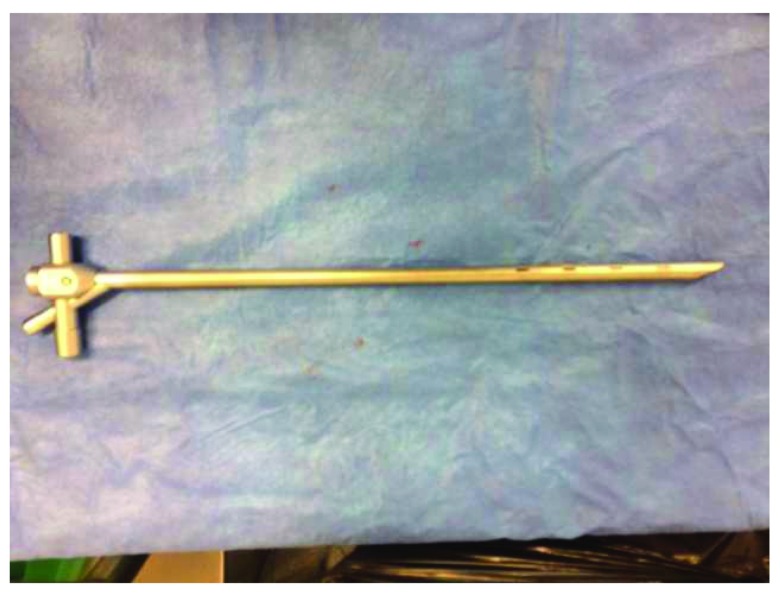
Rigid bronchoscope. This figure is an original image taken in ED’s clinic for this publication.

The first indication for the use of bronchoscopy was foreign body removal. Gustav Killian used a metal pipe to remove a pork bone from a farmer’s lung in 1897. This was done with topical cocaine as a local anesthetic. It was a great advancement at the time, as foreign body aspiration would likely cause infection and lead to death. With advancements in bronchoscopy, it is now possible to use a flexible bronchoscope to remove a foreign body.

A multitude of basket and devices can be used through the working channel of a flexible bronchoscope to assist with foreign body retrieval. Figure 2 represents a Zero Tip grabbing an almond, commonly used type of basket.

**Figure 2.**
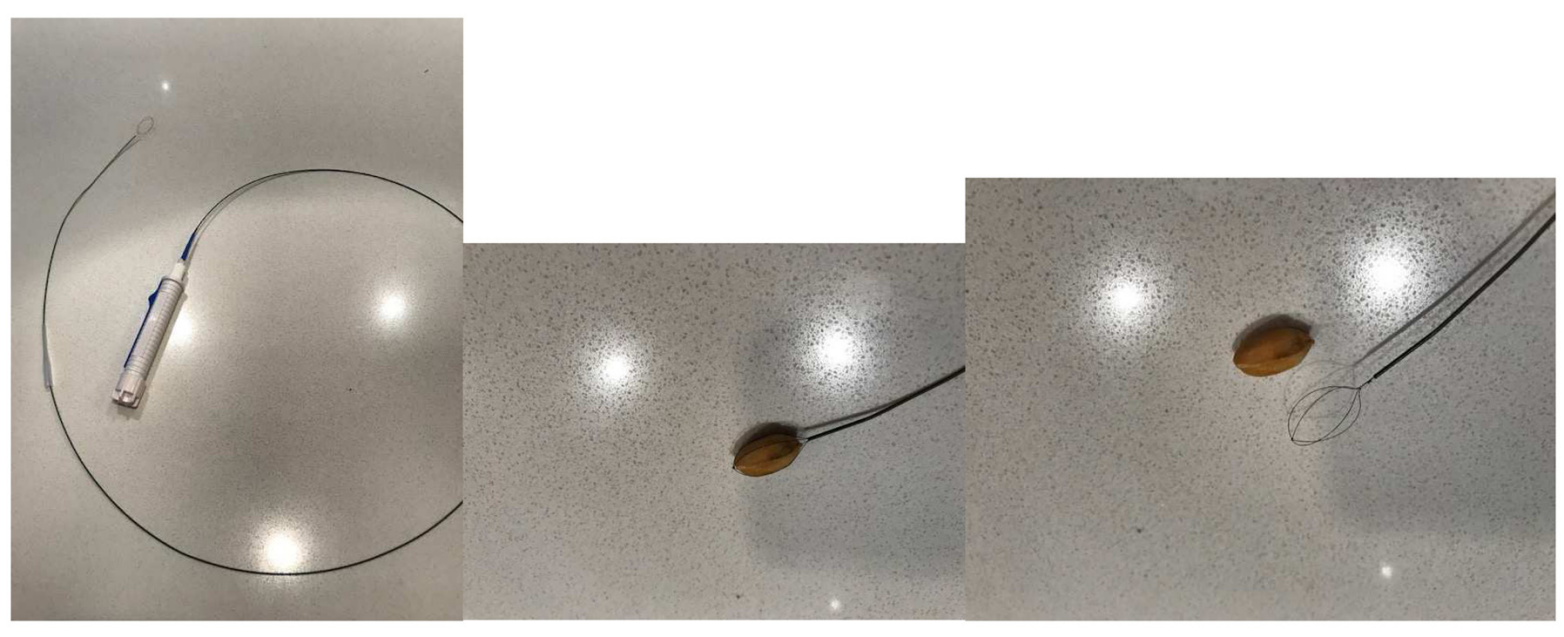
Zero Tip Airway Retrieval Basket (Boston Scientific, Marlborough, MA, USA). **a.** Zero Tip Basket **b.** Almond trapped in Zero Tip Basket **c.** Almond and Zero Tip Basket side by side. This figure is an original image taken in the author’s clinic for this publication.

For vegetable matter, the use of cryotherapy is a good option. Small, flexible cryoprobes are used through the working channel. The freezing probe is applied to the foreign body, which then is removed all at once with the bronchoscope ^1^. The use of a rigid bronchoscope remains indicated for any sharp or metal object at risk of causing bleeding or tearing the mucosa. The main difference between the procedure performed now and that performed in 1897 is that the former is done under general anesthesia in the operating room.

Another common indication of bronchoscopy is for the diagnosis and treatment of lung cancer.

When a flexible bronchoscope is combined with ultrasound technology, it is possible to perform lung cancer staging by gaining access to several lymph node stations in the mediastinum. It also increases the yield for sampling peripheral lung nodule ^2^. There are two different types of ultrasound: the linear probe and the radial probe. The linear probe is helpful for any central lesion; this includes hilar masses, mediastinal lesions, and lymph nodes. Ultrasound allows direct visualization of the lymph nodes as a transbronchial needle aspiration is performed ( Figure 3).

**Figure 3.**
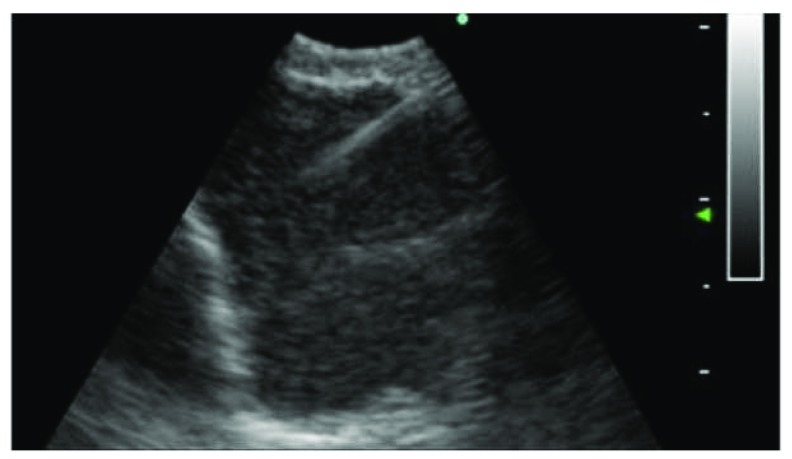
Lymph node sampled with a needle under ultrasound guidance. This figure is an original image taken in the author’s clinic for this publication.

The sample obtained allows diagnosis, staging, and molecular testing for advanced malignancy. This is used for lung cancer but also for metastatic cancer to the lungs from other primaries. Sampling of the lymph nodes can be useful for other diseases such as sarcoidosis and lymphoma and infections such as tuberculosis.

The radial probe is used for peripheral lesions. The combination of a radial probe and a guide sheath allows sampling with biopsy forceps, a cytology brush, and a needle. It increases the diagnostic yield of bronchoscopy from 30 to 70% depending on the studies for peripheral lung nodules ^3^. Not only does it improve the yield but it also has a lower risk of pneumothorax. Lung nodules can be malignant or benign, and obtaining a biopsy can help in determining the best management.

Electromagnetic navigation bronchoscopy (ENB) is another tool to aid in the diagnosis of peripheral lung nodules. The diagnostic yield of ENB varies, depending on the study and the expertise of the operator, from 33 to 96% ^4^. It uses a real-time intrabody navigation system with electromagnetic fields and tracking sensors.

Both endobronchial ultrasound and ENB require a biopsy in order to obtain a diagnosis. A promising innovative technology called optical coherence tomography provides high-resolution imaging close to histology and might avoid the need for a biopsy in the future. This would reduce the risk of bleeding and pneumothorax.

For central endobronchial lesions, bronchoscopy can be used to restore the airway lumen. Multiple tools, including electrocautery, cryotherapy, laser, and mechanical debulking, have been described. Stents, either metal or silicone ( Figure 4), are used to maintain patency once the airway is open. This technique enhances quality of life as a palliative measure in lung cancer ^5^.

**Figure 4.**
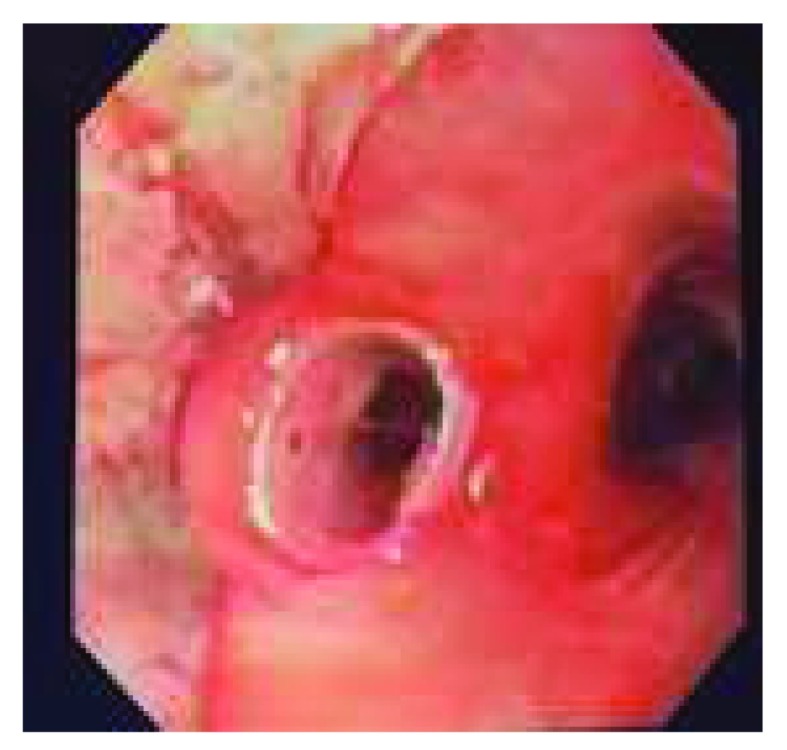
Silicone stent insertion with rigid bronchoscopy for left mainstem bronchus stenosis. This figure is an original image taken in the author’s clinic for this publication.

Some types of low-grade lung cancer, including carcinoid tumor, mucoepidermoid carcinoma ( Figure 5), and adenoid cystic carcinoma, can be cured with the use of bronchoscopy, usually with the use of electrocautery or cryotherapy.

**Figure 5.**
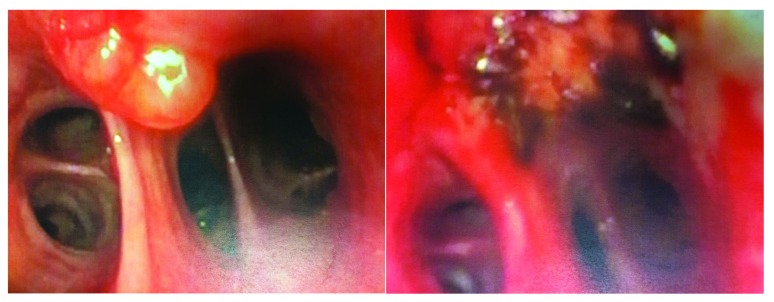
Before (left) and after (right) treatment of a mucoepidermoid carcinoma with electrocautery through flexible bronchoscopy. There was no recurrence at 5 years. This figure is an original image taken in the author’s clinic for this publication.

Some patients will present with massive hemoptysis. The etiology for massive hemoptysis can be diverse, including coagulopathies, vasculitis or other vascular diseases, lung cancer, bronchiectasis, and trauma. It is often a situation that can be stressful for both the patient and the pulmonologist. Bronchoscopy, most commonly rigid bronchoscopy, can be helpful to determine the location of the bleeding and can allow the protection of the airway. Patient ventilation can be maintained through the rigid bronchoscope while the bleeding is concealed. Often, it will not provide a permanent treatment, but it can temporize the bleeding and stabilize the patient until the definitive treatment is applied. Balloons and bronchial blockers are of great help in isolating the bleeding ^6^.

In recent years, the role of bronchoscopy has become more common for the diagnosis of interstitial lung diseases. Cryobiopsies present an advantage over transbronchial biopsies to obtain larger samples. There is still some progress that needs to be made to standardize the procedure. It might not replace the standard of care that is open lung biopsy, but it appears to be a less intrusive, safer, and more accessible procedure ^7^.

In the treatment of chronic obstructive pulmonary disease, bronchoscopy can use valves, coil implants, or thermal vapor ablation to mimic lung volume reduction surgeries. It seems to be safer than the surgical procedure, mostly for being less invasive when patients are well selected. Patients with severe air trapping and thoracic hyperinflation seem to benefit the most from these procedures ^8^.

Another advancement in bronchoscopy has been for the treatment of severe asthma. Bronchial thermoplasty appears to provide a decrease in asthma exacerbations, emergency department visits, and hospitalizations. The idea behind this procedure is to reduce the hyperactivity of the airway by affecting the smooth muscle layer with heat ^9^.

Recently, a new treatment for aspergilloma was developed using bronchoscopy. In the presence of an access point to the cavity from an airway, endoscopic removal of the fungus ball ( Figure 6) and cleaning of the lung cavity with the combination of a rigid and a flexible bronchoscope have been reported to improve patients’ symptoms. This technique has been used for patients who, owing to medical comorbidities, are not candidates for surgery ^10^.

**Figure 6.**
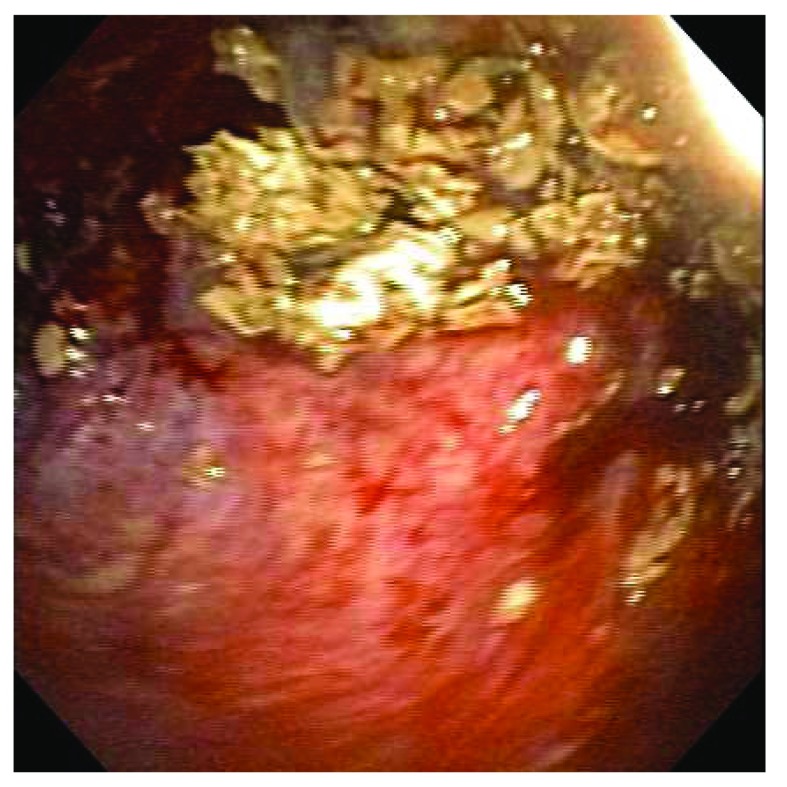
Endoscopic view of an intracavitary aspergilloma. This figure is an original image taken in the author’s clinic for this publication.

Bronchoscopy has improved many lives. It offers a good combination of diagnostic and therapeutic options for a variety of lung diseases. It has led to the new medical field of interventional pulmonary medicine, in which training is oriented toward mastering all of the different bronchoscopic procedures. As the technology improves, there will be new tools and new challenges. Medicine is evolving toward less-invasive, higher-yield approaches with fewer complications, and bronchoscopy is a good example of that progress.
